# A Psychologist-Led Educational Intervention Results in a Sustained Reduction in Neonatal Intensive Care Unit Infections

**DOI:** 10.3389/fped.2014.00115

**Published:** 2014-11-19

**Authors:** Hans Van Rostenberghe, Jacki Short, Noraida Ramli, Tan Beng Geok, Sivasangari Subramaniam, Che Anuar Che Yaakob, Azizah Othman, Nor Rosidah Ibrahim, Jacqueline Ho, Zeehaida Mohamed, Habsah Hasan

**Affiliations:** ^1^Department of Paediatrics, Universiti Sains Malaysia, Kubang Kerian, Malaysia; ^2^School of Public Health, University of Sydney, Sydney, NSW, Australia; ^3^Department of Paediatrics, Penang Medical College, Georgetown, Malaysia; ^4^Department of Obstetrics and Gynaecology, Universiti Sains Malaysia, Kubang Kerian, Malaysia; ^5^Department of Medical Microbiology and Parasitology, Universiti Sains Malaysia, Kubang Kerian, Malaysia

**Keywords:** infection control, psychologist, nurse education, infection rate, neonatal intensive care unit

## Abstract

Even though in the corporate world psychological science has been widely used, the formal use of evidence-based psychology in important areas of clinical medicine has been scanty at best. It was the aim of this study to determine the efficacy of a psychologist-led 2-week nurse educator training on the infection rate in the neonatal intensive care unit (NICU).

**Materials and methods:** In 2007, six senior neonatal nurses underwent a training course focusing on the retrieval of evidence and knowledge of psychological principles that would allow them to share the evidence in such a way that evidence is effectively brought into practice. The course was led by a psychologist. The nurses created and delivered their own teaching modules, all focused on infection control. The rates of bacteremia, 2 years prior to intervention were analyzed and compared with the rate following the intervention for 3 years.

**Results:** The immediate output of the course included three teaching modules: hand washing, sterile procedures, and general measures to control infection. These modules were subsequently administered to the NICU nurses in regular structured continuous nursing education sessions. The psychological techniques taught in the course were applied. Bacteremia in the NICU significantly decreased in the year of the course and the subsequent years when compared to previous years (from more than 17 in 2005 and 2006 to less than 10 per 100 admissions to the NICU in 2008 and 2009).

**Conclusion:** This study suggests that a psychologist-led course, followed by a structured CNE can lead to a sustainable reduction in infection rates in a NICU.

## Introduction

Psychological techniques have been widely used in the corporate world, but reports of formal integration of psychological knowledge into clinical practice and management have been scanty at best. Here, we report an initiative that aimed at the use of established psychological techniques to control the infection rate in a neonatal intensive care unit (NICU).

In many low and middle income countries, including Malaysia, the outcomes for very low birth babies (VLBW) are not as good as in developed nations. Nosocomial infection is one major contributing factor to morbidity and mortality in the neonatal unit. Infection rates in several of the NICUs in Malaysia can be as high as 20–40% for the VLBW (1–3). The infection rate for VLBW ranged from 6 to 62% and for extremely low birth weight (ELBW) from 2 to 36% babies, as evidenced by data collected by the Malaysian National Neonatal Registry (MNNR) in 2006.

Malaysian hospitals tend to have a very high delivery rate (above 10,000 deliveries per year is no exception) but most hospitals are relatively well equipped with modern ventilators, incubators, and monitoring systems. Important treatment modalities are available and the majority of hospitals have neonatologists who are trained overseas for at least 1 year. Most hospitals have policies and protocols similar to those used in developed nations. Reasons for high infection rates may be related to limited infrastructure such as inadequate ward ventilation and inadequate space leading to overcrowding.

The other important cause includes problems with knowledge, attitude, and practice of the staff working in the neonatal units (4, 5). Most Malaysian nurses have graduated from a 3-year diploma program with minimal evidence-based skills training. Recently, the government encouraged degree courses for nurses but up to now, only a minority of nurses have benefited from it. Many nurses in Malaysian NICUs start working without having any advanced neonatal training. Those with advanced training are not actively involved in the decision making of patient management. Many neonatal units have organized continuous nursing education (CNE), but it is not very well-structured, often delivered by doctors, and often poorly planned and infrequently delivered (personal communications).

A well-structured in-service CNE program with an active learning approach, conducted by trained nurse educators could possibly help to improve some of the poor outcomes for VLBW and ELBW infants in Malaysia. While there are reports of temporary successful courses in hand washing, their sustainable effects on attitude and practice seem hard to achieve (6). In order to obtain a more sustainable improvement, a different approach was tested. A 2 week psychologist-led nurse education program was organized in July 2007 as a part of the South East Asia Optimizing Reproductive and Child Health in Developing Nations (SEA-ORCHID) project in cooperation with Hospital Universiti Sains Malaysia (HUSM).

The SEA-ORCHID project was a collaborative, multidisciplinary, international health project designed to increase the health of mothers and babies in nine South East Asian hospitals through evidence-based research generation and implementation. This article reports on a psychologist-led training course of nurse educators and its effect on bacteremia rate in a NICU. The program was designed to optimize motivation and involvement in learning and sustained behavior change in future nurse educators in the NICU of a tertiary hospital in Malaysia, enabling them to induce behavior change in those they trained. The research hypothesis was that such a program could lead to a sustained reduction in infection in the NICU.

## Materials and Methods

A 2-week nurse educator program was developed and run in July 2007 in the NICU at HUSM. HUSM is a teaching hospital and one of two tertiary hospitals located in the state of Kelantan, a predominantly rural state in the north-east of Peninsular Malaysia. During the study period, HUSM provided pediatric surgery and neurosurgery services to neonates but not cardiac surgery. It has a delivery rate of 7000–8000 deliveries per year. About 20% of admissions are related to prematurity but as preterm babies tend to be admitted longer, about half of the ward is dedicated to the care of preterm babies. Yearly admissions of extremely and very low birth weight babies amount to 30–40 and 80–100, respectively. Over the past decade and during the whole study period of the current study, these figures have not undergone major changes. Admissions during the study period varied from 1100 (2005) to 1700 (2009) per year.

During the study period, the admission criteria for babies were not changed and the physical environment of care also did not undergo any major changes. Even so, there was an increase in the number of total admissions over the years. This increase may be related to the establishment of foeto-maternal services in the hospital and not due to an increase in small preterm babies. The mean yearly admission rate for ELBW was 33 babies per year during 2005 and 2006 (before the intervention) and 29 over the 3 subsequent years. For VLBW, it was 75 babies per year pre-intervention and 85 per year post-intervention.

Twelve staff nurses were involved in the program. Six of them were from the neonatal unit and another six from the labor room. The course was led by a psychologist, specialized in education, and who was appointed as an educator for the SEA-ORCHID project. This program was closely based on principles of adult learning theory (7, 8), social learning theory (9, 10), and constructivism (11).

The purpose of this training was to educate this small group of nurses in the following areas:

- searching for and retrieving evidence relevant to current practice concerns (finding the evidence);
- insights in the psychology of inducing and undergoing behavioral change;
- evidence-based principles of adult learning (effectively sharing the evidence);
- support in the design and delivery of workshops to their peers.

After 3 days of interactive class room teaching, the participants went to search for evidence and were asked to design teaching modules that were identified as relevant to current practice concerns with the help of local content experts. The neonatal nurses were divided into three groups of two and each group was responsible for producing one teaching module. Since the main concerns identified by the neonatal nurses were around infection control, each of the neonatal modules was about different aspects of infection control in the NICU. The modules were based on principles of adult learning for which there was strong evidence and were highly interactive. Subsequently, each group of nurses demonstrated their module to each other and received feedback from content experts and from the course leader. Finally, they taught their peers in the ward and received more feedback. The hand-hygiene module was recommending a seven-step procedure. The approach followed the tell-show-do-feedback principle.

Participants kept a personal reflective learning portfolio. They were asked to make a summary of this portfolio and these summaries were analyzed using a grounded theory approach. The participants also completed a pre-and post-knowledge test on evidence-based practice and effective learning techniques. They were given opportunities for reflection on the course.

We analyzed the effect of the 2-week training for the six neonatal nurses, their subsequent development of the neonatal infection control teaching modules, and the dissemination of these modules on neonatal outcomes by examining the bacteremia rate over a 2 year period before, during, and 2 years after the intervention. The main outcome of the training program was measured in terms of the bacteremia rate in NICU, HUSM during the 2 years following the intervention. All infants admitted to the NICU were included in the analysis.

Data were retrieved from the positive blood cultures database in the Medical Microbiology Laboratory and from the admission database of the NICU. Repeated cultures of the same organism from the same patient were counted as one.

The bacteremia rate was calculated based on the number of positive blood cultures per 100 admissions. Statistical analysis was done using SPSS version 12.1 (SPSS Inc, Chicago, IL, USA). Mean bacteremia rate before and after intervention was compared using independent *t*-test and the significant level was set at 0.05.

## Results

The course was attended by six neonatal nurses. During the course, the participating nurses of the NICU created three teaching modules focusing on:

1. hand hygiene;
2. sterile procedures;
3. other measures to control infection in an NICU.

The module on hand hygiene initially highlighted the written hand-hygiene policy in the NICU and the proper technique for hand hygiene in a Power Point presentation. Following that the trainers went on to show the right technique for each procedure (hand washing, hand rubbing with disinfectant solution). Each of the participants in the session was subsequently asked to perform the procedure, and constructive feedback was provided by the trainers using the sandwich technique in which first the good things are mentioned, then the areas for improvement and then the positive outcome that may follow application of the suggested improvements. This approach to teaching procedures is also known as the tell (teach in PowerPoint) – show (demonstrate) – do (performance by trainees) – feedback technique.

The module on sterile procedures followed a similar pattern, giving a short theoretical introduction, followed by the tell-show-do-feedback approach for essential sterile techniques such as wearing sterile gloves and hub care. The third module was about general measures and also involved having a short PowerPoint about important topics including cleanliness in the ward, isolation/cohorting of infected or colonized babies, and brain storming on how the issues at hand could be improved in the local setting.

Each module was interactive and was fully developed by the nurses themselves, which resulted in a solid sense of ownership and enthusiasm during the delivery of the modules.

Emerging themes from the nurses reflective portfolios included: significant increases in confidence in module presentation, knowledge and skill gains in research and training, and positive suggestions and enthusiasm for ongoing training.

Nurses scored higher in the post-test than in the pre-test. The answers to questions regarding attitude also showed that a marked improvement had occurred. The structured feedback given by the participants showed a high level of appreciation of the course content, the course structure, and the way the course was conducted.

The trained educators presented the modules to all NICU staff at the end of the course and repeated their presentation in slightly different formats every 2 months for 1 year. Since then, other modules covering a range of unrelated areas have been created and CNE has become a weekly, well planned, and structured event in the NICU and almost exclusively delivered by nurses.

After the intervention, the psychological principles learned in the course were applied throughout the workplace by doctors and senior nurses. Whenever feedback needed to be given to nurses, a sandwich technique was used (positive–negative–positive) and the number of days, free of nosocomial infection was visibly displayed in the ward. For every 14 consecutive days of no nosocomial infection, a small celebration was organized.

Bacteremia rates in the NICU during the years before and after the program are shown in Table 1. A marked reduction in bacteremia rate was noted from 2007 onward. The mean difference of the total bacteremia rates before and after intervention were significant (*p* < 0.001). The reduction was significant for both Gram-positive and Gram-negative bacteremias. It was sustained for at least 3 years from the beginning of intervention. The yearly bacteremia rates are shown graphically in Table 2.

**Table 1:** **Mean monthly rate of bacteremia before and following intervention of infection control practice in NICU**.

	Pre-intervention (2005–2006), mean (SD)	Following intervention (2007–2009), mean (SD)	Mean difference	95%CI	*P* value
Rate of bacteremia (number of positive blood cultures/100 admissions)	17.5 (4.7)	10.3 (3.9)	7.1	4.9-9.3	0.027
Rate Gram-negative bacteremia (number of Gram-negative cultures from blood/100 admissions)	7.9 (2.8)	3.9 (2.8)	3.9	2.5, 5.5	<0.001
Rate of Gram-positive bacteremia (number of Gram-positive cultures from blood/100 admission)	10.4 (3.9 )	6.2 (2.8)	4.2	2.4, 5.9	<0.001

**Table 2:** **The mean monthly bacteremia rate for each of the study years**.

Year	Mean	95% CI	SD
2005	17.1	14.4, 20.0	4.4
2006	18.1	15.6, 20.5	3.9
2007	12.0	8.7, 15.3	5.2
2008	9.5	7.4, 11.5	3.2
2009	9.0	7.4, 9.6	2.5

The admission rates per year have increased over the years of the study period making the NICU busier (1131, 1191, 1416, 1485, and 1725 for each of the consecutive study years, respectively). There were no changes in admission criteria. Even though the number of admissions increased, the absolute counts of positive blood cultures per year decreased post intervention.

## Discussion

This psychologist-led 2-week intensive training course for senior nurses from the NICU with focus on retrieval of evidence and effective sharing of evidence is a novel approach in infection control. It was followed by a well-structured continuous nurse education, application of the psychological techniques learned, and a sustained reduction in infection rate in the NICU.

Other studies have reported interventions for infection control to have a variable efficacy and sustainability. Targeted interventions on infection control especially hand-hygiene compliance among staff were associated with significant decreases of bacteremia caused by clonally related pathogens, overall mortality, but had no significant effect on the rates of colonization with drug resistant pathogens (12–14).

Nurse educators have an important role to play in sustaining acceptable infection control. Hand-hygiene promotion, guided by health care workers’ perceptions, identification of the dynamics of bacterial contamination of health care workers’ hands, and performance feedback, was effective in sustaining compliance (14). Education on hand hygiene has been shown to improve the compliance to hand hygiene and infection rate (12, 14, 15). An effective delivery and a proper understanding of the evidence are the keys to the success of the education program.

In some developed nations full-time, trained, in-service nursing educators (often Master or PhD level) are available to provide in-service training. Our present nursing career structure does not appear to support this model. Previous attempts in the NICU where the currently reported study was conducted, to obtain a fully trained in-service nurse educator have failed. Opportunities for further studies were made available and selected NICU nurses, who took these up moved away from the clinical setting usually into senior management or education positions in nursing schools. Our study suggests that an alternative model for providing in-service education is to provide clinical nurses with the above combination of skills. We believe that the success of this program lay in the integration of evidence-based practice skills with the understanding of adult learning and of the enablers and barriers to change. If ward-based knowledge and skills in evidence-based practice and education is desirable in nurses, then it could be asserted that the most expedient way of providing this without the subsequent loss of the trained staff, may be the provision of short and practical hospital based courses to train a critical mass of nurses with these skills. In our setting, we found that six trained nurses were able to significantly reduce bloodstream infections. We feel a psychologist with an understanding in adult education was the key person. The fact that this psychologist had some skills in EBP meant that she could design this effective intervention. The input of a psychologist with such qualifications is likely to be beneficial for the future nurse educators to effectively change behavior of the nurses in the NICU.

A weakness of this study is the lack of controls. With time, the bacteremia rate might have improved even without the intervention. But the sharp and statistically significant decrease in infection rate that happened in the NICU in 2007 and was sustained in subsequent years suggests that the described intervention is likely to have contributed to the acceleration of reduction in nosocomial infections in the unit. The sustainability of the effect of the course may be related to the application of sound psychological knowledge and methods in the CNE and nursing management in the neonatal unit (7–11). Another weakness is that some of the bacteria cultured from the blood of patients in the NICU may have been contaminations rather than real infections. However, it is unlikely that the rate of contaminated blood cultures would have differed a lot over the years, since the technique and frequency of blood cultures has not changed in the NICU. Availability of data on hand-hygiene compliance could have made the link between intervention and outcome stronger, but still the timing of the drop in infection rate is suggestive that the intervention was related to it. Previous attempts to educate the staff in the NICU of HUSM had failed to have a sustained drop in infection rates.

If in other centers, similar courses could be shown to be equally effective, the concept of integrating psychology fully into nursing education may be useful for many other middle and low income countries where neonatal nosocomial infection rates are still high. Developed nations that are short of nursing staff may also be interested in the concept.

In conclusion, a 2-week nurse educator course, integrating psychological scientific knowledge, evidence-based education, and evidence-based medicine was conducted in HUSM and resulted in a well organized and structured nursing CNE sustained over the following 2 years. During the 2 years following the course, there was a significant reduction in the bacteremia rate.

## Conflict of Interest Statement

The authors declare that the research was conducted in the absence of any commercial or financial relationships that could be construed as a potential conflict of interest.
